# A review of the clinical introduction of 4D particle therapy research concepts

**DOI:** 10.1016/j.phro.2024.100535

**Published:** 2024-01-10

**Authors:** Barbara Knäusl, Gabriele Belotti, Jenny Bertholet, Juliane Daartz, Stella Flampouri, Mischa Hoogeman, Antje C Knopf, Haibo Lin, Astrid Moerman, Chiara Paganelli, Antoni Rucinski, Reinhard Schulte, Shing Shimizu, Kristin Stützer, Xiaodong Zhang, Ye Zhang, Katarzyna Czerska

**Affiliations:** a Department of Radiation Oncology, Medical University of Vienna, Vienna, Austria; b Department of Electronics, Information and Bioengineering, Politecnico di Milano, Milano, Italy; c Division of Medical Radiation Physics and Department of Radiation Oncology, Inselspital, Bern University Hospital, and University of Bern, Bern, Switzerland; d Department of Radiation Oncology, Massachusetts General Hospital and Harvard Medical School, Boston, MA, USA; e Emory University, Radiation Oncology, Atlanta, USA; f Department of Medical Physics & Informatics, HollandPTC, Delft, The Netherlands; g Erasmus MC Cancer Institute, University Medical Center Rotterdam, Department of Radiotherapy, Rotterdam, The Netherlands; h Institut für Medizintechnik und Medizininformatik Hochschule für Life Sciences FHNW, Muttenz, Switzerland; i New York Proton Center, New York, NY, USA; j Institute of Nuclear Physics Polish Academy of Sciences, PL-31342 Krakow, Poland; k Division of Biomedical Engineering Sciences, School of Medicine, Loma Linda University; l Department of Carbon Ion Radiotherapy, Osaka University Graduate School of Medicine, Osaka, Japan; m OncoRay – National Center for Radiation Research in Oncology, Faculty of Medicine and University Hospital Carl Gustav Carus, Technische Universität Dresden, Dresden, Germany; n Helmholtz-Zentrum Dresden – Rossendorf, Institute of Radiooncology – OncoRay, Dresden, Germany; o Department of Radiation Physics, Division of Radiation Oncology, The University of Texas MD Anderson Cancer Center, Houston, TX, USA; p Center for Proton Therapy, Paul Scherrer Institute, Villigen PSI, Switzerland

**Keywords:** Intrafractional motion, Particle therapy, Motion management, 4D imaging, Adaptive workflows, 4D Treatment Workshop for Particle Therapy, 4D dose reconstruction

## Abstract

**Background and purpose:**

Many 4D particle therapy research concepts have been recently translated into clinics, however, remaining substantial differences depend on the indication and institute-related aspects. This work aims to summarise current state-of-the-art 4D particle therapy technology and outline a roadmap for future research and developments.

**Material and methods:**

This review focused on the clinical implementation of 4D approaches for imaging, treatment planning, delivery and evaluation based on the 2021 and 2022 *4D Treatment Workshops for Particle Therapy* as well as a review of the most recent surveys, guidelines and scientific papers dedicated to this topic.

**Results:**

Available technological capabilities for motion surveillance and compensation determined the course of each 4D particle treatment. 4D motion management, delivery techniques and strategies including imaging were diverse and depended on many factors. These included aspects of motion amplitude, tumour location, as well as accelerator technology driving the necessity of centre-specific dosimetric validation. Novel methodologies for X-ray based image processing and MRI for real-time tumour tracking and motion management were shown to have a large potential for online and offline adaptation schemes compensating for potential anatomical changes over the treatment course. The latest research developments were dominated by particle imaging, artificial intelligence methods and FLASH adding another level of complexity but also opportunities in the context of 4D treatments.

**Conclusion:**

This review showed that the rapid technological advances in radiation oncology together with the available intrafractional motion management and adaptive strategies paved the way towards clinical implementation.

## 1 Introduction

Proton and carbon ion therapy offer advantages when compared to conventional photon therapy concerning tumour coverage and sparing of adjacent organs at risk (OARs). However, due to the steep dose fall-off and the interplay effect between the delivery of the scanned beam and the target motion, particle treatments are much more sensitive to organ and tumour motion and associated range uncertainties than photon therapy. Therefore, the management of interfractional changes and intrafractional motion remain some of the most challenging aspects of particle therapy (PT), especially when pencil beam scanning (PBS) is employed.

Looking back 15 years, leading scientists in the field of PT set ambitious aims for four-dimensional (4D) particle therapy with real-time respiratory motion management that included major developments (e.g., implementation of spot scanning technology for moving targets, clinical use of rescanning, development of beam gating and beam tracking and motion-dependent patient selection guidelines) [1]. Regardless of the exceptionally fast developments in this field, these ambitions have only been partly met today [2], [3], [4], [5], [6], [7], [8], [9].

Modern, individualised radiotherapeutic concepts consider the effects of motion in different ways, depending on the time scale of motion and its impact on the dose delivered to the target and normal tissues. For example, swallowing movements occur infrequently on the time scale of dose delivery, only moderately affecting the treatment of head and neck cancer patients. Conversely, respiratory as well as cardiac motion and peristalsis are constantly present and can have a large impact on the treatment of, e.g., lung, liver, pancreas and oesophagus cancer [10], [11]. In lung cancer patients, the amplitude and pattern of tumour motion are influenced by diaphragm motion, the tumour location as well as the tumour volume and disease stage [12], [13]. Day-to-day changes in bowel position and shape mainly affect the pelvic area (prostate, gynaecological and rectal cancers). Classification of motion patterns into different types (cyclical, continuous drift, erratic and unpredictable) and time scales (from inter- to intrafraction) automatically raises the question of which movements can and should be compensated for in 4D radiotherapy. In addition to the motion-induced uncertainties, setup and range uncertainties, anatomy and tumour changes must also be considered when developing 4D PT treatment concepts [14], [15].

To account for the impact of motion on the dose distribution, many concepts have been developed and implemented over the last few years. In conventionally fractionated radiotherapy, the treatment is spread out over several weeks allowing for an averaging of the impact of motion on the dose distribution. In hypofractionated treatments the averaging becomes less effective due to the limited number of fractions. Moreover, systematic errors can still occur independent of the fractionation scheme but are potentially compensated by robust optimisation or adaptation. Robust optimisation and evaluation have been commonly used in PT to ensure adequate dose to the tumour region and sparing adjacent organs at risk while simultaneously accounting for inter- and intrafractional movements [16]. In clinical practice, the three-dimensional (3D) robust optimisation is well established for static tumour regions prone to interfractional variations. Also, it has been shown that the intrafractional motion might be sufficiently compensated by the internal target volume (ITV) concept, overcoming the necessity of 4D robust optimisation [17], [18]. However, the final decision depends on the individual case and the centres’ technical capabilities.

In the adaptation concepts, changes in the patient’s anatomy over time are mainly accounted for by replanning. It has also been shown that adaptation better preserves target coverage and organs at risk sparing than robust optimisation [19]. From a workflow perspective, offline (within days) and online (within minutes during the treatment fraction) adaptation approaches can be differentiated. However, the feasibility of adaptation depends not only on the time but also on the indication, treatment modality and strategy [20], [21], [22] as well as online imaging capabilities. Especially in particle therapy shortcomings in automatic re-planning and auto-contouring have resulted in a lack of online adaptive strategies [16], [17], [18], [23].

In terms of PT with ultra-high FLASH dose rates even more attention should be paid to the timing of motion and beam delivery potentially changing the complexity of intrafractional motion compensation [24]. In cases when motion compensation is not feasible during initial or adaptive treatment planning, the adequate classification of breathing motion and evaluation of the interplay effect becomes crucial in PT [25], [26], [27], [28], [29], [30], [31], [32], [33]. While organ movements can be predicted theoretically, the timing of data extraction, processing, synchronisation and correlation modelling needs consideration for a time-efficient treatment planning process. Alternatively, the impact of motion can also be reduced by optimizing the spot weight, spot delivery patterns and incident beam directions for fast, time-efficient and robust dose deposition [34], [35], [36].

The goal of this review was to summarise the most relevant and current research findings as well as dynamic developments related to the clinical implementation of 4D PT that are not reflected in traditional long-lasting surveys. Its scope focused on the latest technological improvements, imaging, workflow design, artificial intelligence (AI) and FLASH in the context of the transition of 4D PT from research to clinical application.

## 2 Material and methods

The structure of this review article followed the scientific contributions of the *4D Treatment Workshops for Particle Therapy* held in 2021 and 2022 (Suppl. A1; Table A1) and previous reports of those workshops [2], [3], [4], [5], [6], [7]. The content of these meetings, surveys and guidelines [8], [9], [37], [38], [39], [40], [41], [42], [43] and relevant articles in the field of 4D PT, mainly published within the last 3 years, built the scientific basis for identifying the way towards clinical implementation of selected motion management and 4D treatment strategies. The literature review was based on a PubMed search (status September 2023).

As a basis we provided an overview of the state-of-the-art technological capabilities for intrafractional motion management. Challenges of computed tomography (CT)-based imaging were addressed, highlighting the possible employment of magnetic resonance imaging (MRI) imaging in the future. The aspects of treatment planning, motion mitigation, dose delivery and reconstruction were summarised in light of the currently clinically used techniques. Moreover, as a reflection of the growing community's interest, a dedicated subsection on adaptive approaches was included.

The current status of motion management and mitigation concepts in clinical practice was based on practice pattern surveys and 4D-treatment-related guidelines (Table 1), supplemented by the talks given during the *4D Treatment Workshops for Particle Therapy* in 2021 and 2022 focusing specifically on the local 4D PT procedures at five particle therapy centres (Table 2).

**Table.1:** Surveys and guidelines relating to online and offline adaptation addressing inter- and intrafractional anatomical changes; RRMM = real-time respiratory motion management.

	Surveys/Guidelines	Content	Modality	Publication year	Survey respondents
Surveys	POP-ART RT part I [39]	Active RRMM	photons	2020	200
	POP-ART RT part II [37]	Offline and online replanning, plan libraries	photons	2020	177
	Online MRIgRT [42]	On-line adaptive radiotherapy using MRI guidance	photons	2020	19
	ARPANSA [40]	Active and passive RRMM and 4D imaging	photons	2022	87
	AAPM TG324 [41]	Active and passive RRMM and 4D imaging - related to TG76	photons	2022	651
	POP-ART PT part I [9]	Active RRMM and rescanning	particles	2023	68
	POP-ART PT part II [8]	Offline and online replanning, plan libraries	particles	2023	68

Guidelines	AAPM TG76 [43]	Active and passive RRMM and 4D imaging	photons	2006	–
	AAPM TG290 [38]	Active and passive RRMM and 4D imaging	particles	2022	–

Current 4D PT research and development topics included aspects of particle imaging, AI and FLASH.

## 3 Technological capabilities for motion management

### 3.1 Imaging

Imaging is crucial at many steps of the 4D PT workflow. The most common and well-established modality for 4D treatment planning is respiratory-correlated 4DCT. However, X-ray-based imaging has the drawback that tissues with the same X-ray attenuation may have different relative proton stopping power ratios (SPR) leading to inaccuracies when converting X-ray attenuation coefficients measured in Hounsfield Units (HU) to SPR [44]. The CT or cone beam CT (CBCT) artefacts, especially in the presence of high-density objects, further limit imaging accuracy. However, the use of dual-energy CT for SPR determination has recently presented an improvement [45], [46], [47]. For multi-energy CT applications, photon counting technology might be available for isocentric in-room imaging in the future albeit the intrinsic limits of CBCT [48].

A second limitation of 4DCT is the simplified correlation of an external mono-dimensional surrogate with the 4DCT phases, which does not necessarily represent the daily breathing patterns and anatomy accurately. Moreover, it can cause artefacts in the reconstructed image [49], [50], [51] while promising improvements in mitigating sorting artefacts, predicting time-resolved information and deriving artefact-free CT and CBCTs were made [52], [53], [54], [55], [56], [57]. For offline evaluations, also image-based motion models and dose-variations models showed encouraging results for applications in PT [58], [59], [60], [61], [62].

X-ray imaging is also standard for daily monitoring and subsequent adjustment approaches. Two-dimensional (2D) X-ray imaging, in-room CT or CBCT (with fiducial markers and in combination with external motion monitoring) can be used to define the tumour position [63], [64], [65]. To detect and account for drifts of moving tumours and make adjustments during irradiation, fluoroscopy-based X-ray imaging and external-internal correlation models can be employed [66].

Another imaging modality used for image guidance is MRI, which allows for the acquisition of 4D and 2D time-resolved sequences [67] with improved soft-tissue contrast compared to X-ray-based imaging. Many groups integrated MRI acquisitions with motion modelling techniques to generate virtual 4DCTs and 3D time-resolved data to further investigate intra‐ and interfraction motion variability [61], [68], [69], [70]. In this regard, the implementation of an in-beam MRI in PT could be a possible solution to overcome the lack of accurate 3D X-ray imaging during dose delivery [71]. While the integration of an MRI with a PBS proton beam [72], [73], [74], [75], [76], [77] and real-time treatment adaptation have been demonstrated to be feasible [78], the conversion of MR greyscale volumes to HU or SPR scales for MRI-only particle beam workflows remains challenging [79], [80], [81].

### 3.2 Treatment planning, motion mitigation and dose reconstruction

Motion management strategies in PT can be divided into passive and active techniques, depending on the patient’s breathing or whether the motion and beam delivery patterns are adjusted to each other or not. Passive motion management techniques encompass the ITV or margin-based approaches, (3D/4D) robust planning, rescanning and optimisation of the delivery sequences [34], [82], [83], [84], [85], [86], [87] ideally followed by a robust evaluation which serves for clinical decision-making [88], [89], [90]. Active motion management adjusts the beam delivery to the motion pattern potentially combined with the regulation of the patient’s breathing. An important aspect is that each active strategy (e.g., tracking, abdominal compression, breath-hold or respiratory gating) raises different concerns and special considerations [2], [11], [91], [92]. These include factors like treatment delivery time, staff requirements, commercial support and patient compliance. For most active motion mitigation techniques real-time monitoring of the patient’s motion is essential and can be facilitated by motion surrogate tracking and optical or fluoroscopic imaging. Stopping the motion instead of compensating for it can be performed by voluntary or controlled breath-hold or using a high-frequency percussion ventilation technique with the potential to reduce the healthy lung tissue included in the high-dose volume [93], [94], [95], [96].

The available accelerator technology is a key parameter in assessing the site-specific characteristics for 4D delivery and surveillance. Commonly used proton cyclotrons show a continuous beam extraction time structure and, despite the considerable energy switching times, relatively fast delivery. This is not the case for synchrotrons used in almost all centres treating with particles heavier than protons. Multiple synchrotron spills are needed to deliver the required number of particles per energy layer. The essential prolongation of the beam delivery time by synchrotrons places considerably different demands on the motion mitigation strategies [97].

The 4D dose reconstruction is essential to evaluate the interplay effect and resulting dose deterioration [27], [28], [29], [30], [31], [32], [33], [98]. CT-based analytical dose calculation algorithms might pose a challenge due to heterogeneities causing Bragg peak degradation [99]. Especially in lung tumours Monte Carlo dose calculation accuracy is advantageous. While used in commercial systems for proton dose calculation, clinical carbon dose calculation is mainly performed with pencil beam algorithms. The dynamic 4D dose calculation based on 4DCT phases, also called dose reconstruction, considers the spot distribution according to delivery parameters which includes the delivery time structure and the patient's breathing traces. Such an approach ideally uses time-resolved delivery patterns from log files and breathing information from fluoroscopy or external motion surrogates. However, a simplification by employing a fixed time structure might be beneficial for prospective analysis [28]. For more complicated treatment regimens or research-oriented retrospective analysis methods like spot-shift dose reconstruction [100], dose reconstruction using 4D MRI [69], [101], [102] or dose tracking with real-time input (e.g., combining optical and sparse monoscopic imaging with kV X-rays) [103], [104] might be relevant.

As for any new treatment dosimetric validation is essential and becomes even more challenging when considering the additional temporal uncertainty. Most detectors used in traditional end-to-end tests or quality assurance (QA) procedures are one or two-dimensional. Thermoluminescent dosimeters (TLDs), ionisation chambers or radiochromic films showed also to be suitable for validating 4D treatment delivery, especially when incorporated with moving phantoms and motion surrogates [31], [32], [97], [98], [105], [106], [107], [108], [109], [110]. Regarding the use of 3D dosimetry systems, (commercial) detector arrays for 4D quality assurance, MRI gels, liquid scintillators or developments for prompt gamma detection can be included [98], [111], [112]. Overall, 4D dosimetry relies on moving 3D dosimetry equipment or time-resolved read-out of the detector signals [113], [114].

### 3.3 Adaptive strategies

Consideration of motion in radiation oncology must be performed including different time scales. While intrafractional changes occur in the timeframe of a single fraction, often requiring dedicated motion management strategies, other anatomical changes might occur over the whole course of treatment in the timeframe of several weeks. During such a long period many anatomical changes may lead to clinically relevant discrepancies between planned and delivered dose distribution independently of intrafractional motion. These changes can be tumour shrinkage/growth, patient’s weight gain/loss, or differences in the filling of the stomach, bowel, rectum, bladder and cavities [115]. Thus, adaptive strategies have gained a large interest in the radiotherapy community, not only as stand-alone concepts but also in combination with intrafractional motion management strategies [116], [117]. Especially in PT adaptive solutions are favourable to compensate for the sensitivity of particle range to density variations along the beam path [118]. To date, several surveys related to the topic have been published for photons and particles presenting the diversity of available and used solutions between centres (Table 1).

Based on the timescale on which the adaptation process is performed adaptive approaches can be differentiated into offline and online. For both approaches the applied adaptation concept heavily depends on the available tools and systems at the facilities.

For offline adaptation concepts implemented in many photon and PT centres [8], [37], tailored institutional solutions for specific treatment sites have been developed. Mobile in-room CT was shown to provide adequate acquisition time, geometric accuracy and image quality as well as SPR conversion as a basis for offline replanning and contour propagation [119], [120], [121]. Individual PT offline adaptive procedures can also be triggered by 2D orthogonal X-ray or (synthetic) CBCT in combination with in-house and commercial software tools [122], [123], [124], which are both also relevant image modalities for 4D motion management. Most offline adaptive planning in photon and particle therapy occurs ad-hoc based on anatomical changes noted on control imaging (e.g., CBCT or diagnostic CT scanner installed in or outside the treatment room) or clinical observations by medical doctors, radiation therapy technologists or the patient. If re-computation of the dose on a new CT shows a necessity for plan adaptation, much of the initial planning and QA procedure must be repeated with some semi-automated parts (e.g., transfer of the original contours onto the repeated image set or setup of the optimisation formulation in the TPS).

Currently, online daily radiotherapy solutions are commercially available only for photon therapy with dedicated platforms using MR guidance [42] or AI-guided CBCT-based systems [125]. However, also the field of PT shows broad interest in implementing more online or hybrid approaches, especially including daily replanning and employing plan libraries [8], [22], [126], [127], [128]. Recently, several key players in the field of real-time adaptive PT joined forces [129] to tackle the remaining challenges and pave the way towards online adaptation in PT [130], [131], [132], [133], [134], [135], [136]. These challenges include aspects of dose accumulation, contour propagation, daily plan re-optimisation and approval as well as quality assurance of the online adaptive treatments [137], [138].

## 4 Clinical implementations of 4D PT treatments based on international guidelines

The clinical implementation of 4D PT was facilitated thanks to the growing availability and accessibility of particle systems together with specific technological developments. The transition from research to clinic also built on the experience gained from photon therapy and was supported by international guidelines for photons and particles (see Table 1).

As an example, the ARPANSA and TG324 photon surveys showed that passive respiratory motion management with the ITV concept was more widely used than active methods [40], [41]. Following the AAPM Task Groups TG76 and TG324 survey on motion management for photon therapy [41], [43], the AAPM Task Group report 290 focused exclusively on 4D PT [38]. It summarised passive and active motion management techniques and considered their suitability concerning breathing motion amplitude and technical limitations. Moreover, it provided guidance for commissioning and QA procedures when implementing 4D treatments and related therapy risks. Other site-specific guidelines for 4D particle radiotherapy were published on behalf of the PTCOG thoracic subcommittee and were dedicated to non-small cell lung cancer (NSCLC) and thoracic malignancies in general [36], [139]. While the first addressed the advantages and cost-effectiveness of PT, the second focused on practical aspects of 4D thoracic treatments and technical requirements. The clinical implementation of these recommendations and guidelines (Table 1) was summarised in the most recent surveys on pattern of practice approaching harmonisation of 4D treatment approaches [8], [9], [140]. It has been well understood that respiratory motion management is essential for treatment sites located in the thorax and abdomen with breath-hold gating (for (left-sided) breast cancer) widely implemented in conventional photon therapy. Other active respiratory motion management strategies, like gating using a respiratory surrogate or surface imaging as the main trigger or the synchronisation by tumour tracking are less frequently used [39], [40], [41].

Clinical implementation of a treatment strategy should be supported by a motion management diagram classifying the actions according to the patient’s motion amplitude [38]. Usually, the lesions located in the lower lobe of the lung and liver are characterised by a motion greater than 10 mm, whereas the middle and upper lobe of the lung (5–10 mm) or pelvis and para-aortic nodes lesions exhibit motion typically below 5 mm [13]. A special case is the oesophagus, which usually does not move more than 5 mm, but the motion of surrounding tissues can exceed 10 mm. Even though the employment of a facility-specific motion diagram supports clinical decisions regarding the choice of 4D treatment approaches, it does not automatically harmonise the 4D PT concepts among different centres. Used solutions are very diverse due to the variety of available tools and systems and there is no common solution valid for all centres. To give a better insight into the practical and envisaged clinical implementations, the following paragraph describes examples of the currently designed workflows at selected PT centres while centre-specific internal criteria and used motion management techniques are summarised in Table 2.

**Table 2:** Example of motion management and mitigation solutions clinically implemented at selected particle facilities (FB – free breathing, BH – breath-hold, AVG – average, MIP – maximum intensity projection); *threshold was not provided by the institute.

Facility	Imaging motion assessment	Indication	Motion threshold	Additional motion mitigation used?	Treatment planning/additional motion mitigation	Repainting used?	Interplay evaluation
MD Anderson Cancer Center (MDCC)	FB scan	breast, partial breast	heart outside the treatment field	no	FB scan used for planning	no	4D simulator [141]
			heart inside the treatment field	yes	BH scan (3x)		
		upper lung, lower abdomen	5–10 mm	no	MIP scan for contouring, AVG for planning, 0 and 50 phases reconstructed	yes	
		oesophagus	<5 mm (but surrounding tissues > 10 mm)	no	MIP scan for contouring, AVG for planning, 0 and 50 phases reconstructed, overrides about the diaphragm	yes	
	4DCT + 3D patient’s model (including interior-exterior anatomy)	lung, liver, oesophagus	<10 mm	no	MIP scan for contouring, AVG for planning, 0 and 50 phases reconstructed	no	
			>10 mm	yes	BH scan (3x)	no (if motion < 5 mm)	
						yes (if motion > 5 mm)	

Emory Proton Therapy Center (EPTC)	4DCT	thorax, abdomen	≤5 mm (with or without abdominal compression)	no	–	no	Inh/Exh dose calculation
			>5 mm	yes	BH (3x) with SDX	no	BH (3x) dose calculation
					planning mitigations (4DRO, reduced beam modulation, repainting, increased number of beams, reduced energy layer spacing, degraded spot)	depending on residual effects seen on 4DDD calculation	4DDD

New York Proton Center (NYPC)	4DCT + WETSA (water equivalent thickness statistical analysis) [80]	lung	<5 mm	no	–	no	4D dose calculatio n [142], [143], [144]
			5–10 mm		–	no (If ≥ 3 beams)	
						yes (if < 3 beams)	
			10–20 mm	yes	abdominal compression (for low-lobe cases), spot size enlargement	yes	
			>20 mm	–	not considered for protons	–	
		liver	<5 mm	no	–	no	
			5–10 mm	yes	abdominal compression or DIBH if tolerable	no (If ≥ 3 beams & for 10 or more fractions)	
						yes (if < 3 beams & for 10 or more fractions)	
			10–20 mm	yes	abdominal compression + other mitigation strategy (e.g., DIBH if tolerable, SDX system)	yes	
			>20 mm	–	not considered for protons	–	

Danish Center for Particle Therapy (DCPT)	4DCT 3–4 exhale BH	liver, oesophagus, lung, lymphoma, breast	<10 mm	yes/no	FB (but might be combined with abdominal compression and if gating not feasible)	no	Spot-shift dose reconstruction [66], [100]
			>10 mm	yes	BH, gating		

Massachusetts General Hospital (MGH)	4DCT	liver, oesophagus, cardiac sarcoma	internal*	yes	DIBH, gating (in development)	yes	4D dose calculation

In those centres the common imaging modality used to assess motion characteristics and support further imaging and treatment decisions was 4DCT with additional motion management based on the assessed tumour amplitude and internally established criteria. For targets exhibiting relatively small motion extent (usually below 5 mm, see Table 2) no special motion management technique was applied. However, 4DCT-based plan recalculation was still performed for evaluation purposes. If the amplitude exceeded the agreed thresholds, motion mitigation techniques became necessary although other parameters like tumour size, position and fractionation might influence that decision. The availability and application of motion management techniques varied among the centres and included, for example, abdominal compression systems, breath-hold techniques, the use of a breathing control system, spot size enlargement or repainting. These techniques were also implemented separately or in a combined way to obtain the most reproducible and comfortable treatment position. During the treatment planning stage, further decisions on, e.g., the number of beams or robust optimisation were made. Depending on the assessed motion amplitude, either free breathing, breath-hold or average CT scans were used for treatment planning. For breath-hold the scans were usually repeated three times to ensure reproducibility. Additionally, CT images for the end-inhale and the end-exhale breathing phase were used for dose evaluation purposes. To provide optimal gantry angles some of the centres performed motion evaluation based on water equivalent thickness statistics to minimise the deteriorating effect of target motion [8]. Either 3D or 4D robust optimisation was included at the planning stage to overcome the dose deterioration effects due to respiratory motion. Estimation of the plan quality and target dose degradation due to the interplay effect was further supported using the 4D interplay simulator [141], 4D dose calculation [142], [143], [144] or spot-shifting [100].

## 5 Current research and development themes

### 5.1 Particle imaging for 4D particle therapy

Proton CT was originally proposed by Alan Cormack in 1963 [145] followed by the first paper on proton radiography a few years later [146]. After taking many decades to mature, particle tracking technology has reached a stage where it could be implemented into clinical workflows in the next ten years [147]. In addition to the limitations of X-ray-based planning mentioned in section 3.1, simultaneous beam’s eye view imaging of the treatment area with X-rays while delivering proton therapy is currently infeasible. This additionally underlines the need and potential for proton or ion imaging for planning and in-room verification, particularly in 4D applications [148].

Proton and ion radiography involves a low-intensity proton or ion beam to create 2D images. By measuring the positions and energies of the protons or ions before and after passing through the object it is possible to infer the most likely path of individual particles and their water-equivalent path length (WEPL). Two-dimensional WEPL imaging provides the possibility to verify that the distribution of stopping power is correct from the beam’s eye-view perspective [147].

Proton CT could solve the HU conversion inaccuracy problem providing an accurate pre-treatment verification method operating at very low doses. It takes the principles of proton radiography a step further by reconstructing 3D or 4D images of the object’s SPR, which are practically artefact-free. As in proton radiography, protons are directed to the object from multiple angles. This allows for iterative data reconstruction of SPR from WEPL measurements and most likely path estimations for millions of protons traversing the object. Large efforts were invested in offering research groups access to fast and accurate open-source reconstruction possibilities [149], [150].

Proton CT systems have been developed in research projects over the past two decades [151], [152], [153]. However, the current systems have faced challenges in their clinical implementation due to technical issues in delivering an ultra-low intensity beam consisting of a single proton or ion per radiofrequency bunch but also related to accuracy and resolution [154]. Ideally, if both imaging and treatment beams are used in the same session, they should be mixed or delivered in a rapid sequence. To address this limitation, a new approach has been proposed, which involves a dual ion source generating deuterium ions (d+) and helium ions (He2+) or multi-nucleon ions [155], [156]. Also 4D-tracking detectors are very promising to simultaneously measure the particle position and time with a high spatial resolution [157]. These innovations aim to overcome the technical difficulties of delivering the ultra-low intensity imaging beam and improve the clinical application of particle CT systems but still lack experimental data on phantom or small animal models.

### 5.2 AI-assisted 4D and real-time imaging

Artificial intelligence (AI) has been playing an increasingly important role in the motion management of radiation therapy. Among other applications it could enable 4D real-time tumour localisation through fast 4D imaging. An example method uses a deformation-driven approach to deform a planning 4DCT to on-board 4D-CBCTs under the guidance of limited-angle on-board projections [158], [159]. A motion model is built using principal component analysis to solve CT-to-CBCT deformation vector fields with free-form 2D-3D deformable registration applied to correct residual errors. To compute 2D-3D deformable registration in seconds, compared to several hours of traditional iterative methods, a deep learning-based 2D-3D-ReNet framework was developed. The limited accuracy of a standard 2D-3D deformable registration for low-contrast regions, such as the liver, was introduced by biomechanical modelling to represent the low-contrast liver as a tetrahedral mesh and allows an increase in the tumour’s localisation accuracy [160]. Further imaging speed and accuracy improvement (below 250 ms latency time) can be achieved by introducing a graph neural network-based deep learning MeshRegNet-Bio framework thanks to which low-contrast liver tumour localisation via a single X-ray projection is possible [161]. The model used motion features encoded in a single X-ray projection to solve the liver boundary motion and subsequently fed it into biomechanical modelling for liver tumour localisation with an accuracy below 1.6 mm.

As another example, the construction of dynamic CBCTs using spatial and temporal implicit neural representation (STINR) addressing the inter-scan anatomy and intensity variations (e.g., tumour shrinkage) was proposed [162]. STINR maps the unknown image and its motion into spatial and temporal multi-layer perceptrons and iteratively optimises the neuron weightings via acquired projections allowing the tracking of a lung target to an average centre-of-mass error of 1–2 mm.

AI has made significant progress in 4D real-time imaging and model-based tumour localisation. Aided by the information provided by planning 4DCTs, tissue biomechanics, 4D or dynamic CBCTs the real-time volumetric tumour localisation can be estimated and/or reconstructed within seconds or less. Studies presented at the 4D workshops and ongoing research show the indisputable potential of AI in the 4D imaging and radiotherapy field and its potential to move from a pure research topic to an active area of translational research and development.

### 5.3 FLASH particle therapy

In recent years FLASH radiotherapy, characterised by delivery at ultra-high dose rate (>40 Gy/s), has been heavily investigated not only for photons but also for proton and carbon ions [163]. To observe that effect, a low-oxygen environment is required for photon, electron and proton beams [164]. For carbon beams it has been suggested that the FLASH effect may occur even in the absence of hypoxic conditions and several institutes have started experimental irradiations [165], [166], [167]. For PBS therapy additional considerations for defining the dose rate need to be included since the dose at each point of the field sums up from multiple spots [168], [169]. In terms of 4D radiotherapy, ultra-fast delivery of fields/fractions (<0.1 s) might potentially minimise the aspect of motion during treatment while considering manifold aspects related to dosimetric, temporal and spatial parameters [170]. However, due to the high sensitivity of the dynamic PBS delivery to moving targets [38], the impact of respiratory motion on the proton FLASH delivery and the corresponding motion management for FLASH radiotherapy is largely unclear. An initial investigation of the effects of respiratory motion on the transmission of proton FLASH dose was performed through simulation and moving phantom measurements [171]. The simulation study using clinical-relevant free-breathing respiratory motion and PBS delivery parameters showed a clinically unacceptable degradation of the delivered dose when compared to the static delivery. However, the treatment quality could be restored by gated delivery at the maximal inhalation or exhalation phase. Phantom measurements quantitively confirmed that dose distortions are limited due to ultra-short beam-on time and relatively stable positions at peak phases [172]. Ultra-fast beam delivery would make breath-hold clinically feasible for most patients [173] and with volumetric imaging guidance breath-hold can warrant a static target treatment condition for FLASH radiotherapy. Therefore, breath-hold and free-breathing gated [174] delivery at the extreme phases serve as the potential motion management strategies to ensure the high consistency of the proton FLASH delivery.

## 6 Concluding remarks

The field of 4D PT combines a diversity of research topics and has successfully advanced to be established in clinical practice. Together with the remarkable developments in radiation oncology over the last years the precision of 4D PT could be improved also incorporating a higher level of personalisation during planning and delivery. Despite remaining heterogeneities in current practical applications, surveys and guidelines illustrate the will and need of the community to work towards harmonisation of clinical protocols.

Topics like 4D treatment planning, motion management, dose reconstruction, imaging and adaptive strategies are progressing towards the state-of-the-art. New themes, such as particle imaging, AI-assisted real-time imaging and FLASH emerge as tomorrow’s translational research topics adding another level of complexity to the context of 4D PT.

## Declaration of competing interest

The authors declare that they have no known competing financial interests or personal relationships that could have appeared to influence the work reported in this paper.
