# Supplementary material for: A review of the clinical introduction of 4D particle therapy research concepts
Source: Phys Imaging Radiat Oncol. 2024 Jan 10;29:100535. doi: 10.1016/j.phro.2024.100535 (PMC10828898; doi:10.1016/j.phro.2024.100535)
Supplement: Supplementary data 1 [file mmc1.docx]

Supplementary material

**A1**

Since 2009 *4D Treatment Workshop for Particle Therapy* meetings have taken place on an annual basis. The main purpose of these workshops is to bring together experienced researchers, clinical personnel, as well as PhD students, to share their ideas and expertise. In total, six *4D Treatment Workshop for Particle Therapy* reports were published in different journals over the last 13 years, summarising the current status and the most recent developments in 4D PT [2–7]. In 2021 and 2022 around 120 participants contributed to the workshops that took place in Delft, Netherlands and Houston, USA.

Perspective talks were given by M. Hoogeman and M. van Vulpen (Holland PTC, Netherlands) in 2021 and by E. Koay and R. Zhu (MD Anderson, US) in 2022. Especially in Houston, a full session was dedicated to clinical treatment strategies, which are in detail described in section 4 summarising the presentations given by T. Whitaker and E. Koay (MD Anderson Cancer Center), S. Flampouri (Emory Proton Therapy Center), P. Poulson (Danish Center for Particle Therapy), M. Kang (New York Proton Therapy Center) and O. Zeidan (Orlando Health Cancer Institute). H. Li (Johns Hopkins Medicine) summarised the AAPM TG290 guidelines. Imaging aspects were presented by C. Paganelli (Politecnico di Milano) and D. Low (University of California), accompanied by a debate on “MR vs X-ray guided proton therapy” between A. Hoffmann (Helmholtz Zentrum Dresden Rossendorf) and D. Schaart (TU Delft). The view on the role of artificial intelligence (AI) in this context was given by Y. Zhang (UT Southwestern Medical Centre). Additionally, to clinical treatment strategies for intra-fractionally moving targets, S. Korreman (Danish Center for Particle Therapy) and J. Daartz (Massachusetts General Hospital) gave an insight into the daily adaptation strategies for proton therapy, while J. Bertholet (Insel Spital Bern) bridged the gap to photon therapy. The look into the future of FLASH for moving targets was presented by B. Rothwell and M. Lowe (Manchester Cancer Research Center), H. Lin (New York Proton Center) and S. Shimizu (Hokkaido University) even touching important aspects of carbon ions. Further, R. Schulte (Loma Linda University) presented the role of particle imaging for moving targets, N. Peguret (Hirslande) introduced strategies for stopping the tumour motion and H. Palmans (MedAustron) gave insights into 4D dosimetry. Both workshops were completed by a roundtable discussion on treatment planning strategies and motion mitigation techniques. Out of the many valuable contributions in the form of posters, the six best contributions from A. Duetschler, F. Lebbink, T. Pan, M. Oud, and V. Maradia were awarded.

Following the growing interest in 4D PT and online adaptive strategies among the radiotherapy community, the 14^th^ edition of the *4D Treatment Workshop for Particle Therapy* will take place at the Paul Scherrer Institute in Switzerland in 2023 and in Aarhus, Denmark in 2024.

Table A1: 4D Treatment Workshop for Particle Therapy Program 2021 and 2022

| **Delft, The Netherlands** | | |
| --- | --- | --- |
| **Title** | **Speaker** | **Institute, Country** |
| Clinician's perspective on 4D proton therapy | Marco van Vulpen | Holland PTC, The Netherlands |
| Physicist's perspective on 4D proton therapy | Mischa Hoogemann | Holland PTC, The Netherlands |
| Learning from MD Anderson experience -  how to manage moving targets? | Thomas J Whitaker | MD Anderson, US |
| Do we currently see what we treat? | Chiara Paganelli | Politecnico di Milano, Italy |
| MR vs X-ray guided proton therapy -  which way to go (Debate) | Aswin Hoffmann | Helmholtz Zentrum Dresden Rossendorf,  Germany |
|  | David Leibold (replacing Dennis Schart) | TU Delft, The Netherlands |
| Motion management for indications  beyond the thorax region | Per Poulsen | Danish Center for Particle Therapy, Denmark |
| Dosimetry in 4D | Hugo Palmans | MedAustron, Austria |
| How to stop a tumor that moves with respiration? | Nicolas Peguret | Hirslande, Switzerland |
| Hot to get FLASH moving? | Beth Rothwell Mat Lowe | Manchester Cancer Research Center, UK |
| How to do daily adaptation in proton therapy? | Stine Korreman | Danish Center for Particle Therapy, Denmark |
| 4D treatment planning: round table discussion | All |  |
| **Houston, Texas, United States** | | |
| **Title** | **Speaker** | **Institute, Country** |
| Clinical perspective on 4D proton therapy | Eugene Koay | MD Anderson, US |
| Physicist's perspective on 4D proton therapy | Ron Zhu | MD Anderson, US |
| Clinical implementation of the treatment of moving targets (overview from different centres) | Heng Li | Johns Hopkins Medicine, US |
|  | Minglei Kang | New York Proton Center, US |
|  | Stella Flampouri | Emory Proton Therapy Center, US |
|  | Omar Zeiden | Orlando Health Cancer Institute, US |
| ART - from offline to online to real-time | Jenny Bertholet | Insel Spital Bern, Switzerland |
|  | Juliane Daartz | Massachusetts General Hospital, US |
| Potential of particle imaging for 4D particle therapy | Reinhard Schulte | Loma Linda University, US |
| 4D motion mitigation techniques:  round table discussion | All |  |
| Beyond 4DCTs: It's about time | Daniel Low | University of California, US |
| AI-assisted 4D and real-time imaging for radiotherapy treatment planning and delivery | You Zhang | UT Southwestern Medical Centre, US |
| Impact of respiratory motion on proton  PBS FLASH radiotherapy | Haibo Lin | New York Proton Center, US |
| Motion management for the carbon ion beam  therapy and FLASH beam delivery | Shinichi Shimizu | Hokkaido University, Japan |
